# Application and Mechanism of Action of Carvacrol Against *Aspergillus niger* Causing Postharvest Rot of Garlic Scapes (*Allium sativum* L.)

**DOI:** 10.3390/jof11100709

**Published:** 2025-09-30

**Authors:** Pei Li, Wenqing Wu, Can He, Boxi Tan, Shijing Tang, Lu Yu

**Affiliations:** 1 Guizhou Key Laboratory of Miao Medicine, Qiandongnan Engineering and Technology Research Center for Comprehensive Utilization of National Medicine, Kaili University, Kaili 556011, China; 2 School of Liquor and Food Engineering, Guizhou University, Guiyang 550025, China; wuwenqing0809@163.com (W.W.); 18212045070@163.com (C.H.); 18090952291@163.com (B.T.); 17585204647@163.com (S.T.)

**Keywords:** carvacrol, *Aspergillus niger*, inhibitory activity, CWI signaling pathway, MAPK signaling pathway

## Abstract

During prolonged storage of garlic scapes (*Allium sativum* L.), the proliferation of microorganisms, particularly fungi, frequently results in postharvest rot, which negatively impacts both product quality and market value. Carvacrol, a promising natural food preservative, exhibits broad-spectrum bioactivity against various microorganisms. In this study, a specific pathogenic fungal strain causing postharvest rot in garlic scapes, designated as HQ, was initially isolated from symptomatic garlic scapes. Based on a combination of physiological characteristics and molecular identification techniques, the HQ strain was identified as *Aspergillus niger*. Our findings further demonstrated that carvacrol exhibits significant in vitro inhibitory effects against *Aspergillus niger* with an EC_50_ value of 75.99 μg/L. Moreover, scanning electron microscopy (SEM) observations revealed that carvacrol induces irreversible morphological and structural changes in the hyphae, resulting in deformation and rupture. Additionally, integrated transcriptomic and proteomic analyses indicated that carvacrol primarily targets the cell wall integrity (CWI) signaling pathway within the mitogen-activated protein kinase (MAPK) signaling pathway in *Aspergillus niger*, thereby compromising cell membrane integrity and stability, which ultimately suppresses fungal growth and proliferation.

## 1. Introduction

Garlic scapes (*Allium sativum* L.), the flower stalks that emerge as garlic plants begin to bolt following vernalization, are native to much of Asia and North Africa, particularly China, and are now cultivated globally [1]. These scapes are characterized by their crisp yet tender texture and high juice content, and they are rich in cellulose, vitamin C, allicin, polysaccharides, minerals, and other essential nutrients [2]. Recently, the consumption of garlic scapes has increased due to their recognized nutritional benefits [3,4]. With growing consumer preference for convenient, fresh-cut produce, garlic scapes are expected to become an increasingly valuable agricultural commodity [1].

During prolonged storage periods of food products, the proliferation of microorganisms frequently leads to postharvest rot, characterized by moss-like growths on stem lesions, tissue softening and decay, surface depressions, and, in severe cases, structural breakage—collectively contributing to a significant decline in commercial value [5,6]. Utilizing natural products derived from plants with inherent disease resistance to manage both pre- and postharvest diseases offers a promising and innovative strategy within the framework of sustainable agricultural development [7,8]. This method presents a safer alternative to conventional chemical agents, as it demonstrates reduced toxicity toward natural predators, humans, and other mammalian species.

Previous studies have demonstrated that carvacrol, a phenolic monoterpene compound containing a free hydroxyl group and naturally present in the essential oils of various plants, possesses a wide range of biological activities, including antimicrobial, antioxidant, and anticancer effects [9,10]. Owing to its flavoring and antimicrobial properties, carvacrol is predominantly utilized for controlling phytopathogens and postharvest fungal decay in agricultural products, serving as a natural food preservative [11,12]. Furthermore, it has been classified by the U.S. Food and Drug Administration (FDA) as generally recognized as safe (GRAS) and is currently used in the food industry as a Category B synthetic flavoring agent [13,14].

In this study, a specific pathogenic fungus strain was isolated from the symptomatic garlic scapes produced in Guizhou Province, China and identified using a combination of conventional identification method and molecular analysis technique. Additionally, the inhibitory effect and mechanism of action of carvacrol against the specific pathogenic fungal strain was investigated utilizing the combined transcriptome and proteome analysis.

## 2. Materials and Methods

### 2.1. Sample Collection

To identify the specific pathogens responsible for postharvest rot in garlic scapes in China, a total of approximately 300 symptomatic garlic scapes, produced in Guizhou Province, were collected from various vegetable markets on 26 July 2023.

### 2.2. Pathogen Isolation and Molecular Characterization

#### 2.2.1. Pathogen Isolation

Small tissue segments from the infected basal, stem, and apical regions of symptomatic garlic scapes were surface-sterilized using 75% (*v*/*v*) ethanol and subsequently rinsed three times with sterile distilled water. The sterilized samples were then placed onto potato dextrose agar (PDA) medium (comprising 6 g of potato powder, 20 g of glucose, 20 g of agar, and 1 L of sterile distilled water) and incubated at 28 °C for 72 h. Hyphae emerging from the tissue explants were aseptically transferred to fresh PDA plates using an inoculation loop and further incubated at 30 °C for 48–72 h. Individual hyphal colonies were selected and subcultured onto new PDA plates twice to ensure microbial purity. The purified fungal cultures were then maintained at 4 °C for subsequent analyses.

#### 2.2.2. Pathogenicity Test

The pathogenicity of the isolated specific pathogen was evaluated by inoculating a conidial suspension (1.0 × 10^6^ conidia/L) onto the basal, stem, and apical regions of 20 fresh garlic scapes using an inoculating needle. Following a 7-day incubation period at 28 °C with 95% relative humidity, rot symptoms similar to those observed in the original symptomatic samples developed on the basal, stem, and apical sections of the garlic scapes. The pathogen was successfully re-isolated from the infected tissues, fulfilling Koch’s postulates, and was subsequently subjected to further characterization through morphological analysis and DNA sequencing [15].

#### 2.2.3. Morphological Characterization

After 72 h of incubation on PDA plates, the morphological characteristics of the specific pathogen were examined using a scanning electron microscope (SEM; Hitachi Ltd., Tokyo, Japan). Briefly, hyphal samples were fixed with 2.5% glutaraldehyde at room temperature for 24 h, subsequently rinsed three times with 0.1 M phosphate buffer (15 min per rinse). Following this, the samples were post-fixed in 1% osmium tetroxide (OsO_4_) solution for 1 h. The specimens were then dehydrated through a graded ethanol series (20%, 50%, 80%, and 100%), with each concentration applied for 5 min. After critical point drying and gold sputter coating, SEM imaging was performed to observe the hyphal morphology of the specific pathogen [16].

#### 2.2.4. Molecular Biological Characterization

Approximately 25 mg of the specific pathogen was collected for genomic DNA extraction using the TIANamp Fungal DNA Kit (Tiangen Biotech Corporation Limited, Beijing, China). The DNA concentration and quality were assessed using an ASP-3700 spectrophotometer (ACTGene, Piscataway, NJ, USA). Molecular identification was performed by sequencing the ribosomal DNA internal transcribed spacer (ITS) region with primers ITS1 (5′-TCCGTAGGTGAACCTGCGG-3′) and ITS4 (5′-TCCTCCGCTTATTGATATGC-3′), as well as the translation elongation factor 1-alpha (TEF-1α) gene using primers EF1 (5′-ATGGGTAAGGAGGACAAGAC-3′) and EF2 (5′-GGAAGTACCAGTGATCATGTT-3′) [17,18]. The PCR amplicons were sequenced by Sangon Biotech (Shanghai, China) and submitted to the National Center for Biotechnology Information (NCBI, https://www.ncbi.nlm.nih.gov/ (accessed on 13 May 2024 and 20 May 2024, respectively)) database under accession numbers PP767448.1 and PP792772.1, respectively. Sequence similarity analysis of the isolates was conducted using the Standard Nucleotide BLAST program (v2.17.0) against the NCBI database. A phylogenetic tree based on the ITS and TEF-1α sequences was constructed using the neighbor-joining method in MEGA version 11.0 software with 1000 bootstrap replications [19].

### 2.3. In Vitro Antifungal Activity Test

The inhibitory activity of carvacrol against a specific pathogen at various concentrations (25, 50, 75, 100, 125, and 150 μg/L) was evaluated using the mycelium growth rate method [20]. Different quantities of carvacrol were dissolved in 1 mL of DMSO and subsequently mixed with 9 mL of 0.1% Tween 20 solution and 90 mL of PDA medium. The resulting mixture was divided into three Petri dishes and allowed to solidify at room temperature to prepare the PDA plates. Mycelial fragments of the pathogen, approximately 0.4 cm in diameter, were excised from the culture medium and transferred aseptically to the center of each PDA plate using a sterile inoculation needle. The inoculated plates were then incubated at 28 °C for 4 days. DMSO alone was used as the negative control. The inhibition rates of carvacrol at each concentration were calculated using a previously established method [20,21]. The median effective concentration (EC_50_) values were determined using GraphPad Prism 8.3.0 Software (GraphPad, San Diego, CA, USA). All experiments were performed in triplicate.

### 2.4. Effect of Carvacrol on the Hyphae Morphology

The specific pathogen was cultured on a PDA plate supplemented with an EC_50_ concentration of carvacrol for 72 h, while the pathogen treated with DMSO served as the negative control. SEM observations on the hyphae morphology of the specific pathogen were conducted [16].

### 2.5. Transcriptome and Proteomics Analysis

The specific pathogen was cultured on a PDA plate supplemented with an EC_50_ concentration of carvacrol (referred to as HX), whereas the pathogen treated with DMSO was used as the negative control (referred to as HC). After 72 h of incubation at 28 °C, hyphae from both HX and HC samples were harvested for transcriptome and proteomics analyses.

Transcriptome sequencing of the hyphae was performed by Hangzhou Lianchuan Biological Co., Ltd., (Hangzhou, China), using the Illumina HiSeq™ 2000 platform (Illumina Inc., San Diego, CA, USA). To obtain high-quality reads, low-quality sequences were filtered using cutadapt software (v1.9.3), and high-quality clean reads were aligned to the reference genome using HISAT2 software (v2.0.4) [22]. Differentially expressed genes (DEGs) were identified using an R package (v4.5.1) with a significance threshold of *p* < 0.05 and –log_10_(qValue) > 1 [23].

Proteomics analysis of the hyphae was conducted by Guizhou University (Guizhou, China). The raw data were processed using a LC–MS/MS system (5600 Triple TOF MS; AB SCIEX, Foster City, CA, USA) interfaced with a Nano-Liquid Chromatography system (Eksigent, Dublin, CA, USA), and protein quantification was performed with MaxQuant software (version 1.5.8.3) [24,25]. Differentially expressed proteins (DEPs; expression level > 2.0-fold, *p* < 0.01) were identified from the Uniprot database (http://www.uniprot.org/ (accessed on 25 May 2024)) [26].

Gene Ontology (GO) annotations, encompassing biological processes (BP), cellular components (CC), and molecular functions (MF), as well as Kyoto Encyclopedia of Genes and Genomes (KEGG) pathway enrichments for the DEGs and DEPs were performed at http://www.geneontology.org/ (accessed on 25 May 2024) and http://www.genome.jp/kegg/ (accessed on 25 May 2024) pathway, respectively [26].

## 3. Results

### 3.1. Pathogen Isolation and Pathogenicity Test

**Figure 1:**
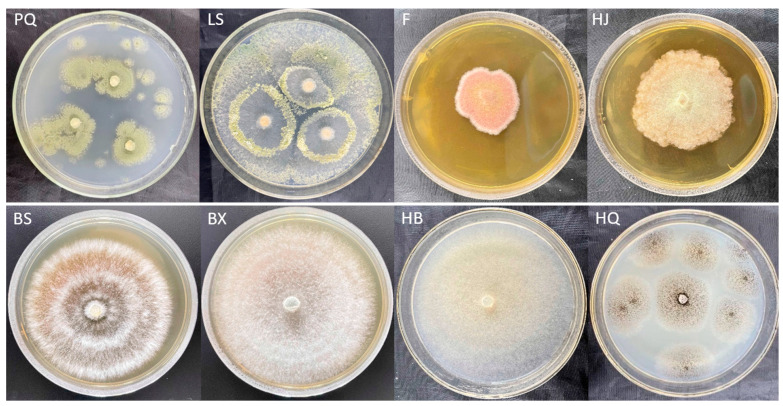
Observe surface of PQ, LS, F, HJ, BS, BX, HB, and HQ strains isolated from the infected base, stem, and apical tissues of symptomatic garlic scapes.

**Figure 2:**
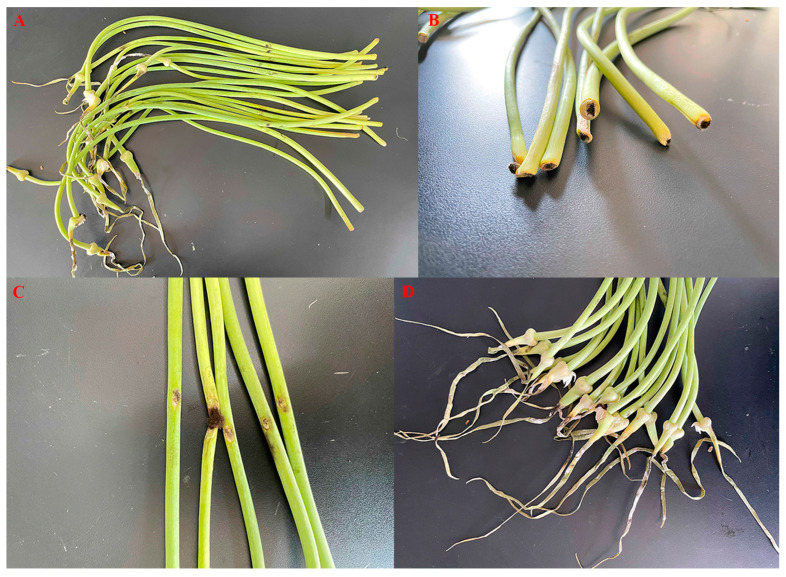
(**A**) Symptomatic garlic scapes collected from vegetable markets; Symptoms of the basal (**B**), stem (**C**), and apical (**D**) regions of garlic scapes after inoculation with HQ strain.

In this study, as illustrated in Figure 1, a total of eight morphologically distinct fungal strains, designated as PQ, LS, F, HJ, BS, BX, HB, and HQ, were isolated from the infected basal, stem, and apical tissues of symptomatic garlic scapes. To evaluate their pathogenicity, pathogenicity tests were conducted on these eight fungal isolates by inoculating a conidial suspension (1.0 × 10^6^ conidia/L) onto the basal, stem, and apical regions of twenty fresh garlic scapes of the cultivar “Chaohua.” Following a 7-day incubation period at 28 °C with 95% relative humidity, as shown in Figure 2, rot symptoms infected by HQ strain resembling those observed in the collected samples were observed in the base, stem, and apical of garlic scapes.

### 3.2. Fungal Identification

**Figure 3:**
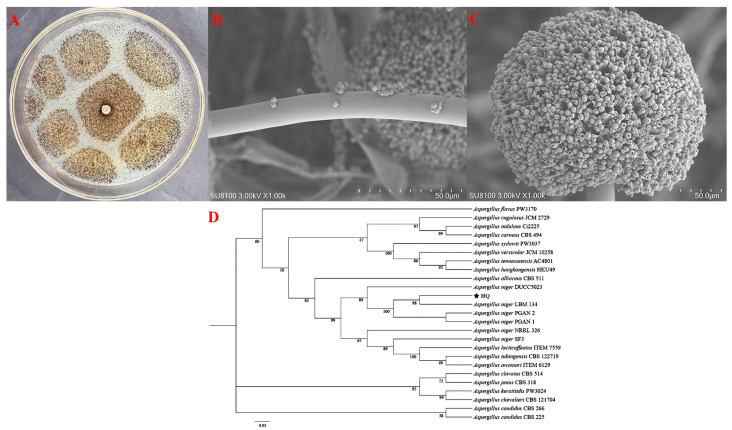
(**A**) Observe surface of HQ strain incubation on front side of PDA medium; (**B**) Morphology of HQ strain mycelia; (**C**) Morphology of HQ strain conidia; (**D**) Phylogenetic tree analysis based on the ITS and TEF-1α resulting sequences of HQ strain.

The HQ strain, re-isolated from symptomatic garlic scapes following Koch’s postulates, was selected for further characterization through morphological and molecular biological methods. Figure 3A shows that, during the early growth stage, the HQ strain exhibited smooth colony margins with thin, soft, outward-extending filaments resembling cotton wool. In contrast, during later growth stages, the mycelium darkened and became easily dispersed. Meanwhile, SEM analysis revealed that the mycelium was elongated and septate with branched structures. The conidial head was spherical, displaying brown to black pigmentation, while the apical sac also exhibited a spherical morphology (Figure 3B,C). In addition, a phylogenetic tree based on ITS and TEF-1α gene sequences was constructed using the neighbor-joining method in MEGA version 11.0 software, showing a 99% sequence similarity between the HQ strain and *Aspergillus niger* (GenBank accession number: MK457457.1) (Figure 3D). Based on molecular identification and distinct morphological characteristics, the HQ strain was conclusively identified as *Aspergillus niger*.

### 3.3. In Vitro Antifungal Activity of Carvacrol Against Aspergillus niger

As shown in Table 1, the inhibition rates of carvacrol against *Aspergillus niger* increased significantly in a dose-dependent manner, reaching 5.86%, 17.71%, 51.45%, 60.66%, 85.23%, and 94.93% at concentrations of 25, 50, 75, 100, 125, and 150 μg/L, respectively. Furthermore, the EC_50_ value of carvacrol against *Aspergillus niger* was determined to be 75.99 μg/L, indicating its potent in vitro antifungal activity against this fungal pathogen.

### 3.4. Effect of Carvacrol on the Hyphae Morphology of Aspergillus niger

**Figure 4:**
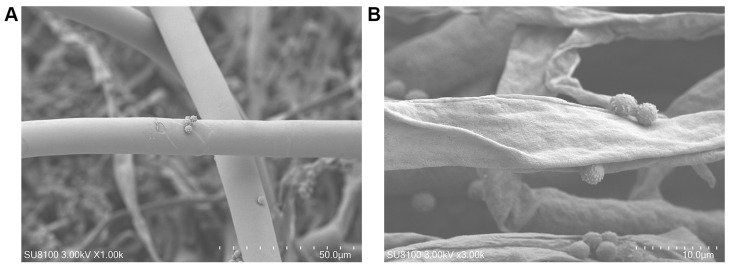
SEM observations on the hyphae morphology of *Aspergillus niger* treated by DMSO (**A**) and carvacrol (**B**).

In this study, SEM was utilized to examine the effect of carvacrol on the hyphal morphology of *Aspergillus niger*. As shown in Figure 4A, the hyphae in the control group displayed a smooth, intact surface with a regular and well-preserved physiological structure. In contrast, Figure 4B shows that the hyphae in the treatment group exhibited significant morphological changes, including irregular contractions, pronounced surface folds, depressions, and shriveled regions. These results suggest that carvacrol induced irreversible structural damage to the hyphae, resulting in deformation and disruption of the cell surface, thereby exerting an inhibitory effect on the growth of *Aspergillus niger*.

### 3.5. Quality Check of Transcriptome Sequencing Data

Table 2 shows that after cleaning and quality checking, the cDNA libraries from HC and HX samples yielded 42.24, 44.64, and 39.32 Mb, and 56.33, 70.13, and 58.69 Mb, respectively. The Q30 base content (base quality > 30) ranged from 93.17% to 94.22%, and the GC content ranged from 54.00% to 55.34%. Overall, the sequencing results are of high quality and the data are suitable for subsequent bioinformatics analysis.

### 3.6. DEGs Identification and Bioinformatics Analysis

**Figure 5:**
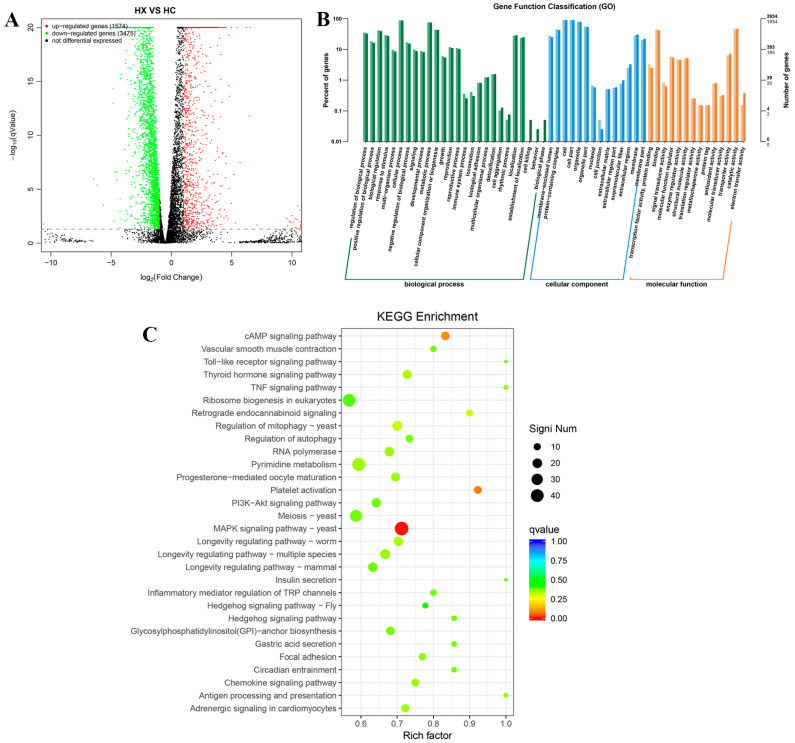
DEGs identification and bioinformatics analysis. (**A**) Volcano plot diagram of DEGs of HX vs. HC. The red points are significant up-regulated genes, the green points are significant down-regulated genes, while the black points are not differential expressed genes. (**B**) Go term enrichment analysis of DEGs of HX vs. HC. (**C**) Top thirty KEGG pathways enrichment of DEGs of HX vs. HC.

As shown in Figure 5A and Table S1, Compared sample HX with HC, a total of 5049 DEGs (including 1574 up-regulated and 3475 down-regulated genes) were detected.

To further functional characterization of the DEGs of HX vs. HC, GO analysis was classified and annotated into 8371 known GO terms, comprising 66.93% (5603 GO terms) in BP, 12.56% (1051 GO terms) in CC, and 20.51% (1717 GO terms) in MF (Table S2). Go term enrichment analysis of HX vs. HC (Figure 5B) demonstrated that the main BP involved regulation of protein modification process, positive regulation of biological process, biological regulation, response to stimulus, multi-organism process, cellular process, negative regulation of biological process, signaling, development process, metabolic process, cellular component organization or biogenesis, growth, reproduction, reproductive process, immune system process, locomotion, biological adhesion, multicellular organismal process, detoxification, cell aggregation, rhythmic process, localization, establishment of localization, cell kinging, behavior, and biological phase. The main CC involved membrane-enclosed lumen, protein-containing complex, cell, cell part, organelle, organelle part, nucleoid, cell junction, extracellular region part, supramolecular fiber, extracellular region, and membrane. The main MF involved transcription factor activity, protein binding, binding, signal transducer activity, molecular function regulator, enzyme regulator activity, structural molecule activity, translation regulator activity, metallochaperone activity, protein tag, antioxidant activity, molecular transducer activity, transporter activity, catalytic activity, and electron transfer activity.

To further functional characterization of the DEGs of HX vs. HC, pathway analysis based on the KEGG database was classified and annotated into 235 known KEGG pathways (Table S3). KEGG pathways analysis of HX vs. HC (Figure 5C) revealed that DEGs were mainly annotated into MAPK signaling pathway, Ribosome biogenesis in eukaryotes, cAMP signaling pathway, PI3K–Akt signaling pathway, TNF signaling pathway, and Toll-like receptor signaling pathway.

### 3.7. DEPs Identification and Bioinformatics Analysis

**Figure 6:**
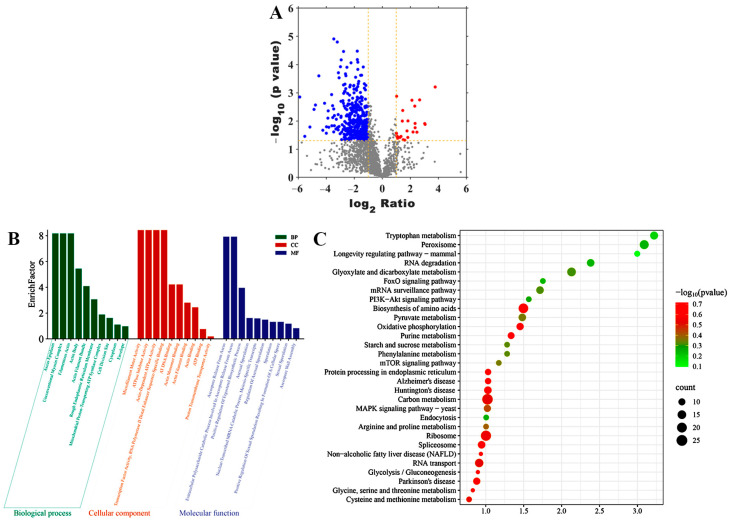
DEPs identification and bioinformatics analysis. (**A**) Volcano plot diagram of DEPs of HX vs. HC. The red points are significant up-regulated proteins, the blue points are significant down-regulated proteins, while the gray points are not differential expressed proteins. (**B**) Go term enrichment analysis of DEPs of HX vs. HC. (**C**) Top thirty KEGG pathways enrichment of DEPs of HX vs. HC.

As shown in Figure 6A and Table S4, a total of 2425 proteins were identified and the up- and down-regulated proteins in HX vs. HC were 363 and 25, respectively.

To further functional characterization of the DEPs of HX vs. HC, GO analysis was classified and annotated into 10,344 known GO terms, comprising 64.96% (6719 GO terms) in BP, 11.45% (1184 GO terms) in CC, and 23.59% (2441 GO terms) in MF (Table S5). Go term enrichment analysis of HX vs. HC (Figure 6B) demonstrated that the main BP involved extracellular polysaccharide catabolic process involved in ascospore release from ascus, positive regulation of ergosterol biosynthetic process, asexual sporulation, nuclear-transcribed MRNA catabolic process, meiosis-specific transcripts, regulation of asexual sporulation, sporulation, positive regulation of sexual sporulation resulting in formation of a cellular spore, sexual sporulation, and ascospore wall assembly. The main MF involved microfilament motor activity, ATPase inhibitor activity, actin-dependent ATPase activity, transcription factor activity, RNA polymerase II distal enhancer sequence-specific binding, AT DNA binding, actin monomer binding, actin filament binding, actin binding, ATP binding, and proton transmembrane transporter activity. The main CC involved ascus epiplasm, unconventional myosin complex, filamentous actin, actin body, actin filament bundle, rough endoplasmic reticulum membrane, mitochondrial proton-transporting ATP synthase complex, cell division site, cytoplasm, and envelope.

To further functional characterization of the DEPs of HX vs. HC, pathway analysis based on the KEGG database was classified and annotated into 309 known KEGG pathways (Table S6). KEGG pathways analysis of HX vs. HC (Figure 6C) revealed that DEPs were mainly annotated into MAPK signaling pathway, Tryptophan metabolism, Peroxisome, Biosynthesis of amino acids, and Carbon metabolism.

### 3.8. Combined Analysis of Transcriptomic and Proteomic Technology

**Figure 7:**
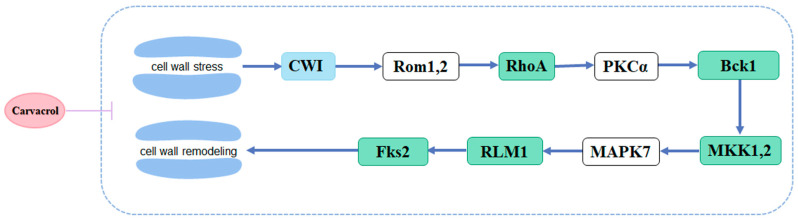
A model of the CWI signaling pathway in *Aspergillus niger* response to carvacrol.

Through integrated analysis of transcriptomics and proteomics, it was found that the number of co-expressed up-regulated and down-regulated genes and proteins was 3 and 127, respectively (Table S7). Within the cell wall integrity (CWI) signaling pathway, a component of the MAPK signaling pathway, ras homolog gene family, member A (RhoA), bypass of C kinase (BCK1), mitogen-activated protein kinase kinase 1 (MKK1), Leptosphaeria maculans 1 (RLM1), and 1,3-β-glucan synthase (Fks2) were all down-regulated. This suggests that carvacrol interferes with the CWI signaling pathway within the MAPK signaling pathway of *Aspergillus niger*, thereby modulating its cellular wall stress response and ultimately leading to structural damage (Figure 7).

## 4. Discussion

Carvacrol is a monoterpenic phenol synthesized by a wide variety of aromatic plants, such as thyme and oregano [9,10]. Currently, carvacrol is utilized in low concentrations as a flavoring agent and preservative in food products, contributing to the extension of shelf life and improvement of microbial safety in perishable foods, including fermented peppers, fruit juices, and fresh-cut fruits [27]. In this study, carvacrol demonstrated strong in vitro inhibitory activity against *Aspergillus niger*. These findings are corroborated by numerous previous studies. Zhang et al. [28] reported that carvacrol may serve as a promising alternative to conventional fungicides for the control of *Botrytis cinerea*-induced gray mold in horticultural commodities. Šimović et al. [29] found that carvacrol exhibited significant inhibitory effects against foodborne pathogens such as *Aspergillus carbonarius* and *Penicillium roqueforti*, thereby enhancing the microbial safety of fresh-cut watermelon.

In the present experiment, SEM observations indicate that carvacrol treatment caused irreversible changes in the morphology and structure of the hyphae, resulting in deformation and rupture. This effect has been previously reported to be associated with damage to the cell membrane of *Botrytis cinerea* and *Rhizopus stolonifer* [28,30].

The mechanism of action of carvacrol against *Aspergillus niger* was investigated through integrated transcriptomic and proteomic analyses. The results indicated that carvacrol primarily affected the CWI signaling pathway within the MAPK signaling pathway in *Aspergillus niger*, which plays a crucial role in regulating cell proliferation and apoptosis, as well as other physiological processes [31,32]. RhoA, a key component of the CWI pathway, is known to play a central role in cell polarity and cell wall biogenesis [33]. BCK1 is an essential element of the CWI pathway involved in maintaining cell wall integrity and preventing fungal cell lysis [34]. MKK1, a mitogen-activated protein kinase within the CWI pathway, is implicated in the regulation of multi-stress responses, potentially through cross-talk with the high-osmolarity glycerol (HOG) pathway in budding yeast [35]. The reduced expression of the transcription factor RLM1 suggests that the transcriptional activity of genes associated with cell wall repair is inhibited. RLM1 is responsible for mediating the majority of the transcriptional output of the CWI pathway [36]. The downregulation of Fks2 leads to decreased synthesis of 1,3-β-glucan, a key structural component of the fungal cell wall, thereby impairing cell wall integrity and its protective barrier function [37]. Our results show that carvacrol down-regulates the expression levels of RhoA, BCK1, MKK1, RLM1, and Fks2. This indicates that carvacrol interferes with the CWI signaling pathway within the MAPK signaling pathway of *Aspergillus niger*, thereby modulating its cell wall stress response and ultimately causing structural damage, which is consistent with the results obtained from SEM analysis.

## 5. Conclusions

In this study, a specific pathogenic fungus strain causing postharvest rot of garlic scapes, designated as HQ, was first isolated from the symptomatic garlic scapes. Our findings also revealed that carvacrol demonstrates significant inhibitory activity against *Aspergillus niger*. In addition, SEM observations indicated that carvacrol could induce irreversible alterations in the morphology and structure of the hyphae, leading to deformation and rupture. Furthermore, integrated transcriptomic and proteomic analyses indicated that carvacrol primarily targets the CWI signaling pathway within the MAPK signaling pathway to interference compromises the integrity and stability of the cell membrane consequently suppressing the growth and proliferation of *Aspergillus niger*. The incorporation of carvacrol as a food preservative can effectively inhibit the growth and proliferation of microorganisms responsible for postharvest decay, thereby enhancing preservation efficacy and extending the shelf life of fruits and vegetables in the postharvest food industry.

## Figures and Tables

**Table 1:** The in vitro antifungal activity of carvacrol against *Aspergillus niger*.

Concentration (μg/L)	Inhibition Rate (%)	EC_50_ (μg/L) ^1^
25	5.86 ± 0.65	75.99 ± 2.31
50	17.71 ± 1.32	
75	51.45 ± 2.02	
100	60.66 ± 2.06	
125	85.23 ± 1.98	
150	94.93 ± 2.34	

^1^ Experiments were performed in triplicate, mean ± standard deviation (SD).

**Table 2:** Overview of transcriptome sequencing date.

Samples	Raw Reads (Mb)	Clean Reads (Mb)	GC Content (%)	Clean Reads ≥ Q30 (%)
HC-1	44.18	42.24	55.04	93.17
HC-2	46.68	44.64	55.27	93.33
HC-3	41.03	39.32	55.34	94.22
HX-1	63.06	56.33	54.70	93.52
HX-2	74.14	70.13	54.00	94.03
HX-3	78.79	58.69	54.39	93.42

## Data Availability

The raw data supporting the conclusions of this article will be made available by the authors upon request.

## Supplementary Materials

The following supporting information can be downloaded at: https://www.mdpi.com/article/10.3390/jof11100709/s1, Table S1: List of DEGs of HX vs. HC. Table S2: The classified and annotated GO terms of DEGs of HX vs. HC. Table S3: KEGG pathways enrichment of DEGs of HX vs. HC. Table S4: List of DEPs of HX vs. HC. Table S5: The classified and annotated GO terms of DEPs of HX vs. HC. Table S6: KEGG pathways enrichment of DEPs of HX vs. HC. Table S7: The number of co-expressed up-regulated and down-regulated genes and proteins of combined analysis of transcriptomic and proteomic technology.
